# Longitudinal Observation of Micromotion upon Loading of Implant–Abutment Connection

**DOI:** 10.3390/bioengineering11060582

**Published:** 2024-06-07

**Authors:** Kohei Yamashita, Yu Kataoka, Motohiro Munakata, Kikue Yamaguchi, Myu Hayashi, Daisuke Baba

**Affiliations:** 1 Department of Implant Dentistry, Showa University Graduate School of Dentistry, 2-1-1 Kita-senzoku, Ota-ku, Tokyo 145-8515, Japan; dtykimp@dent.showa-u.ac.jp; 2 Department of Dental Education, Showa University School of Dentistry, 1-5-8 Hatanodai, Shinagawa-ku, Tokyo 142-8555, Japan; 3 Department of Biomaterials and Engineering, Showa University School of Dentistry, 1-5-8 Hatanodai, Shinagawa-ku, Tokyo 142-8555, Japan; 4 Department of Implant Dentistry, Showa University School of Dentistry, 2-1-1 Kita-senzoku, Ota-ku, Tokyo 145-8515, Japan; munakata@dent.showa-u.ac.jp (M.M.); kyamaguchi@dent.showa-u.ac.jp (K.Y.); 5 Department of Oral Health Management, Division of Oral Function Management, Showa University School of Dentistry, 2-1-1 Kita-senzoku, Ota-ku, Tokyo 145-8515, Japan; myu.h@dent.showa-u.ac.jp; 6 Department of Dental Laboratory, Showa University School of Dentistry, 2-1-1 Kita-senzoku, Ota-ku, Tokyo 145-8515, Japan; baba19860319@cmed.showa-u.ac.jp

**Keywords:** implant loading, micromotion, peri-implantitis, implant–abutment connection, removal torque

## Abstract

While technological advances have made implants a good treatment option with a good long-term prognosis, peri-implantitis, which results in alveolar bone resorption around implants, has been observed in some cases. Micromotion at the implant abutment connection can cause peri-implantitis. However, the temporal progression of micromotion upon loading remains unclear. Therefore, we aimed to longitudinally measure micromotion upon loading application on an implant. Implants with Morse-tapered connections were prepared. Custom titanium abutments were fabricated and tightened onto implant bodies at 35 N. A 100 N vertical load was applied for 200,000 cycles. Micromotion was measured when the load was applied, as was the total implant length and removal torque before and after loading. The micromotion was measured from the position data of the jig of the testing machine during loading. The average removal torque was 30.67 N after 10 min of tightening and 27.95 N after loading, indicating a decrease due to loading. The implant length reduced by 3.6 μm under the load. The average micromotion was 0.018 mm at 2 cycles, 0.016 mm at 100,000 cycles, and 0.0157 mm at 200,000 cycles, indicating implant length reduction under the load but not reaching 0. The micromotion between the implant and abutment under a cyclic load decreased over time but did not completely cease. These results highlight the relationship between micromotion and loading, underscoring the importance of careful monitoring and management to mitigate potential complications, such as peri-implantitis, and ensure optimal performance and durability of the implant.

## 1. Introduction

Many oral implants comprise three pieces, including the bone-anchored titanium (Ti) implant body and a connection platform that performs the transition from the hard tissue region to early osseointegration. International studies have reported a high average success rate of 98% for implant insertion and osteointegration based on the insertion of more than 1,200,000 implants [1,2]. Moreover, recent long-term studies on dental implants have reported high survival rates (82–100%) after an observation period of more than 10 years [3].

Oral implant treatment is increasingly being used to restore oral function. In particular, the introduction of new implant surfaces and implant–abutment connection (IAC) designs has improved dental implant outcomes [4,5]. However, marginal bone resorption around the tooth neck at the site of oral implant placement can result in clinical complications. Marginal bone resorption significantly affects long-term stability, even when osseointegration is achieved. The possible causes include peri-implantitis, surgical trauma, occlusal overload, microleakage, compromised biological width, and reduced-quality alveolar bone [6]. Furthermore, IAC behavior has attracted attention as a possible cause of peri-implantitis. Peri-implantitis is most likely to occur within 1 year of placement of the final prosthetic device. These findings highlight the importance of implant maintenance.

Carlos et al. reported that in the micro-space of the implant–abutment interface (IAI), oral bacterial flora could grow and cause inflammation in the surrounding alveolar bone [7]. Improving the precision of the space in the IAI at the level of the bone crest may reduce the IAI micro-space, thereby reducing the infiltration of inflammatory cells around the implant and preventing marginal bone resorption [8,9,10]. However, there are limitations to the degree of IAI adaptation, and completely eliminating the micro-gap at the IAC is challenging. The micro-gap serves as a factor for bacterial microleakage, increasing micromotion during loading. Furthermore, both micromotion and microleakage contribute to fretting wear, plastic deformation, and screw loosening. Such mechanical damage escalates the micro-gap and micromotion, leading to an exacerbating cycle of microleakage and mechanical damage [9,10].

Methods of assessing the micromotion at the IAI include optical microscopy, scanning laser microscopy, scanning electron microscopy, and different X-ray applications, such as microcomputed tomography and synchrotron-based radiography [11]. No significant correlation was found between the average size of these spaces measured through scanning electron microscopy and the rate of leakage measured through microbiological testing [9].

Despite the clinical importance of micromotion at the IAI, no universally valid method for quantifying this phenomenon has been described. Therefore, the maintenance interval has historically been determined based on clinical reports. Furthermore, to our knowledge, no studies have observed the temporal progression of micromotion when subjected to loading. In this study, the behavior of abutments under function was observed using a universal material testing machine to quantify micromotion over time.

The present study obtained several results by measuring micromotion during loading of implants over time.

- The results revealed trends in micromotion, which is a suspected cause of peri-implantitis.
- This finding may help to elucidate the causes of peri-implantitis and establish prevention and treatment methods.

The method we used to measure micromotion, the measurement results, and the relationship between micromotion and peri-implantitis predicted from the results are described below.

## 2. Materials and Methods

### 2.1. Implant Body and Ti Custom Abutment

**Figure 1:**
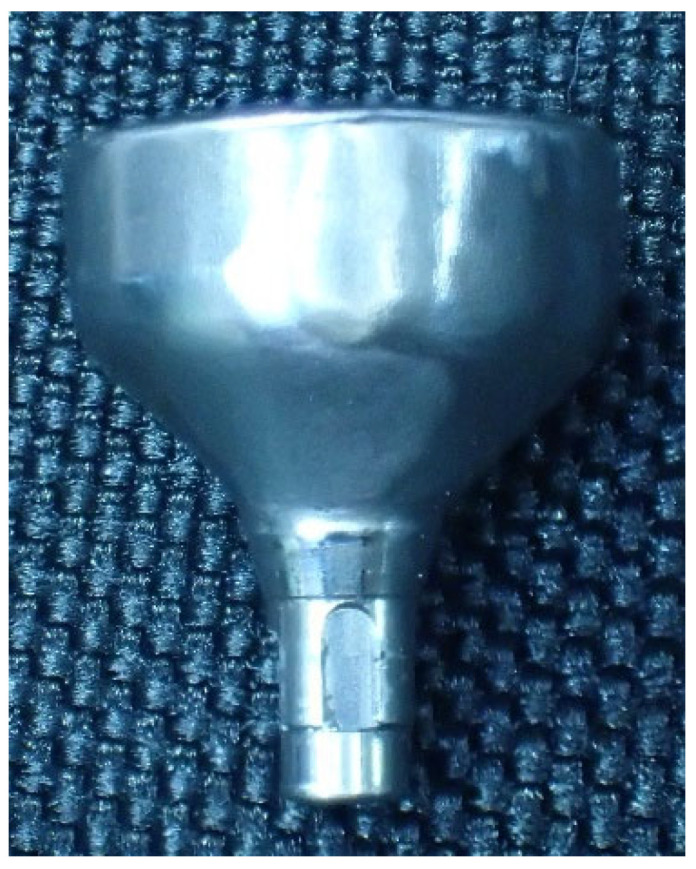
Custom abutment manufactured by Straumann CARES^®^ for single crowns.

Implant bodies with a morphology of bone-level internal tapered connections (Bone Level Tapered φ4.1 10 mm RC SLA^®^ Ti Loxim^®^, Straumann, Basel, Switzerland) were used. A custom-made Ti abutment (RC CARES^®^ Abutment, Straumann) (Figure 1) was obtained according to accepted standards. The abutment was tightened to the implant using the original Ti abutment screw. Five samples were prepared for the study.

### 2.2. Fixation to Measuring Jig

**Figure 2:**
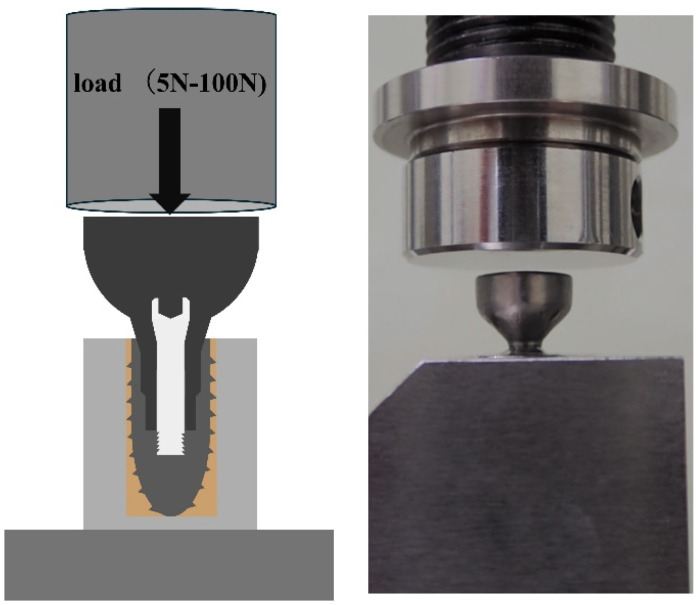
Specimen fixed to the jig using resin cement.

The Ti abutment was tightened to the implant with an original Ti abutment screw at the manufacturer’s specified torque of 35 Ncm and again at 35 Ncm 10 min after tightening, after which the specimen was used. The specimens were fixed with resin cement (Rilayex™ Unisem 2 Automix, 3M, St. Paul, MN, USA) to a special loading jig for implant dynamic fatigue testing according to ISO 14801 (Figure 2).

### 2.3. Fatigue/Durability Test

The specimen was placed in a fatigue/durability testing machine (ElectroPuls E1000, Instron, Norwood, MA USA), such that a load was applied perpendicular to the implant axis. The test conditions were set using a dedicated fatigue testing machine operating software package (Instron CONSOLE, Instron). One cycle involved applying a load of 100 N at a ramp speed of 600 N/s and unloading it to 5 N, for 200,000 cycles under the same loading and unloading conditions. The data acquisition rate was 100 Hz and the position data were mechanically calibrated for each cycle.

### 2.4. Micromotion

**Figure 3:**
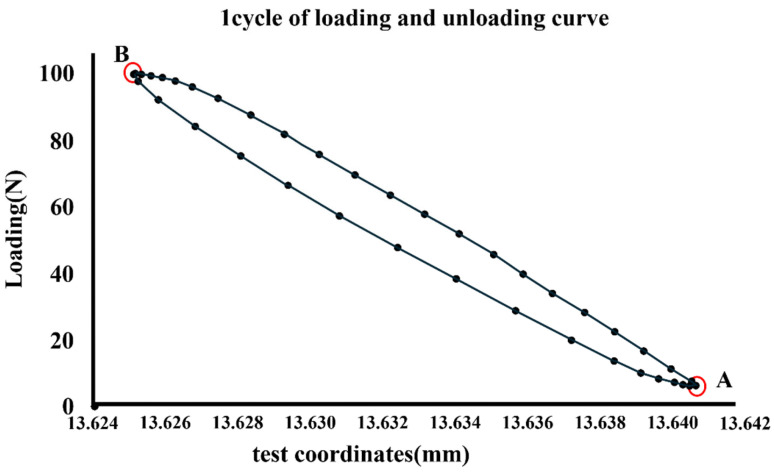
Numbers with the highest and lowest positional information in a cycle designated A and B, respectively.

The obtained position data were analyzed using dedicated collection and analysis software (Instron Wave Matrix, Instron). The numbers with the highest and lowest positional information in a cycle were designated A and B, respectively (Figure 3). The period between the upper and lower ends was considered the micromotion value, and time-lapse data were obtained for up to 200,000 cycles. Micromotion values at 2,100,000, and 200,000 cycles were used for the analysis.

### 2.5. Measurement of Removal Torque

Measurements were performed using a torque-measuring device (Labo Torque Driver, KTC, Kyoto Kikai Tool Co., Ltd., Kyoto, Japan) 10 min after tightening the Ti abutment to the implant body and after a 200,000-cycle load/unload test.

### 2.6. Measuring the Total Length of the Implant and Abutment Assembly

**Figure 4:**
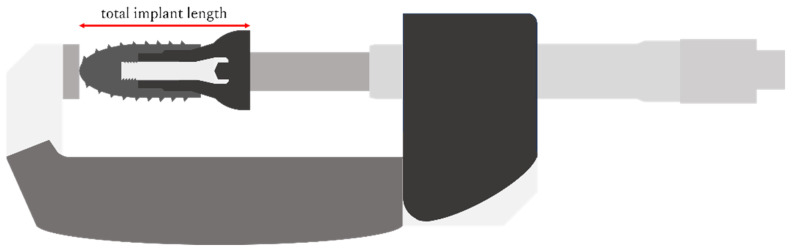
Total length of the implant measured using an electronic digital micrometer.

The total length of the implant–abutment assembly was measured after tightening to 5 N, 10 min after tightening to 35 N, and after the load test using an electronic digital micrometer (Digimatic Outside Micrometer, Mitutoyo, Kawasaki, Japan) (Figure 4).

### 2.7. Statistical Analyses

JMP Pro 17 (JMP Pro 17, Chicago, SAS Institute Inc., Cary, NC, USA) was used for statistical analysis. A one-way analysis of variance and Tukey’s HSD method were used to statistically compare the removal torques of the five implant abutments before and after loading. The same method was also used in micromotion to statistically compare the results at 2, 100,000, and 200,000 cycles. All statistical tests were performed at a significance level of 5%.

## 3. Results

The total length of the implant decreased by an average of 22.2 μm when comparing the tightening torque at 5 N to that at 35 N. Additionally, when comparing 35 N to post-loading, the length decreased by an average of 3.6 μm.

**Figure 5:**
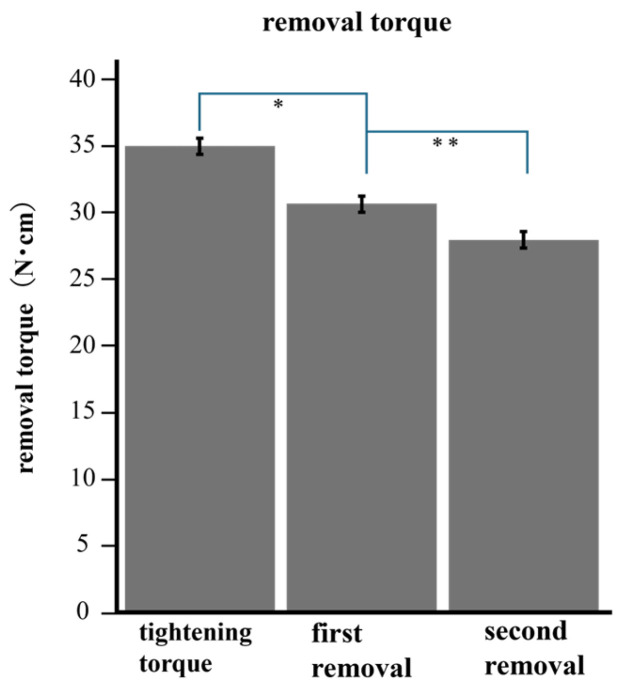
The torque after loading was significantly lower than the removal torque before loading. * (*p* < 0.001) ** (*p* < 0.001).

The removal torques before and after loading are summarized in Figure 5. The mean removal torque was 30.67 N after 10 min of tightening and 27.95 N after loading. After 200,000 cycles, the removal torque was significantly lower than the tightening torque after 10 min (*p* < 0.05).

**Figure 6:**
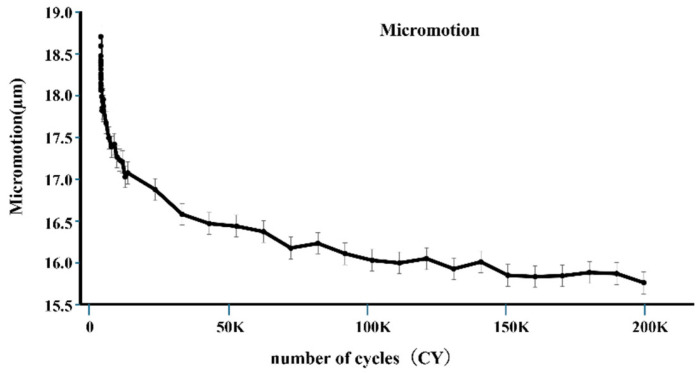
The vertical axis represents the average value of micromotion, and the horizontal axis represents the number of cycles. At the initial stage, micromotion significantly decreased.

**Figure 7:**
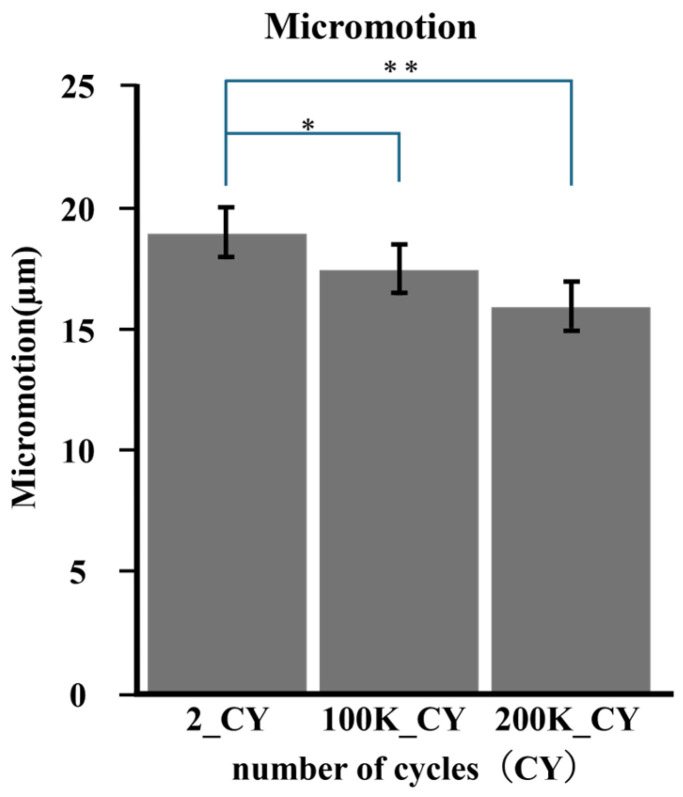
The average micromotion was calculated according to number of cycles. Comparisons were made for each cycle number based on the average value. * (*p* < 0.005); ** (*p* < 0.005).

Micromotion tended to decrease with increasing load cycles, as shown in Figure 6. The mean of micromotion was 0.018 mm at 2 cycles, 0.016 mm at 100,000 cycles, and 0.0157 mm at 200,000 cycles. The micromotion of 100,000 cycles was significantly smaller than that of 2 cycles (*p* < 0.05), and that of 200,000 cycles was also smaller than that of 2 cycles (*p* < 0.05) (Figure 7).

Compared to the 5 N tightening, the total implant length after 35 N tightening and 200,000 cycles of loading was shorter in all specimens. The difference in settlement between 5 N and 35 N was greater than that between 35 N and after loading.

These results indicate that loading causes a decrease in tightening torque, resulting in a smaller micromotion value. We also found that the vertical of the abutment varied with tightening torque.

## 4. Discussion

In the present study, the total implant length tended to decrease after loading. Similar results were obtained in experiments using implants with different Morse-tapered connections for measuring the change in the total implant length with tightening torque and the total implant length after loading in the vertical direction [12]. Implants with a Morse-tapered connection mechanism may allow the abutment to settle into the implant by applying a load perpendicular to the implant axis.

In a study by Kim et al., the abutment screw was re-tightened at least twice with a torque of 30 N cm at 10 min intervals during all clinical examinations and procedures to minimize the loss of initial pressurization [12]. In the present study, load testing was performed after the second tightening at 35 N cm; therefore, it was not necessary to consider the reduction in pressure that occurs in the early stages of screw tightening.

Micromotion and settlement can lead to fewer implant failures in the external system. Moreover, microleakage under load over time can be significantly reduced using tapered connection systems [13,14]. In these studies, the systems were Morse-tapered connection systems and had a structure similar to the implants used in our experiment [13,14]. However, in the present study, micromotion was measured and not microleakage. Measurement of microleakage serves as an indicator of the quantity of bacteria infiltrating the IAI and leaking out of the IAI. Furthermore, measuring micromotion allows for the assessment of the risks of mechanical stimulation to the surrounding tissues of the IAC, as well as fretting wear and plastic deformation that occur in the IAI. In an experiment by Karl et al., micromotion was measured for several implant–abutment combinations with ten loading cycles. A wide range of micromotion values was observed, indicating that no implant shoulder geometry or manufacturing technique completely eliminated micromotion [11]. In our experiment, 200,000 loading cycles were applied; the magnitude of micromotion showed a decreasing trend over time with cyclic loading. However, the micromotion did not reach zero. This indicates that micromotion at the IAC always occurred after the superstructure was placed on the implant body. The results of this experiment indicate that micromotion of more than 10 µm is expected to persist.

Micromotion can lead to numerous issues such as peri-implantitis. Although the bacteria responsible for peri-implantitis are not fully known, Ito et al. observed the presence of bacterial flora specific to the condition [15]. The average size of the bacteria is presumed to be a width of 0.2–1.5 µm and a length of 1–10 µm [16,17]. Micromotion larger than these dimensions may act as a pump to move bacteria in and out of the implant. Therefore, there is a risk of peri-implantitis at any time after superstructural placement. Moreover, recent studies have reported that micromotion and fretting wear can break down the Ti passive film, exposing Ti to the action of the surrounding complex media and resulting in the generation of large amounts of debris and continuous release of Ti ions [18]. A correlation was observed between the release of Ti particles and peri-implantitis [19]. Micromotion in the IACs can be a risk factor for peri-implantitis in terms of both bacterial and Ti-ion leakage.

The results of this study indicate that clinically, occlusal adjustment over time after superstructure placement is necessary because loading causes settlement of the superstructure. Furthermore, regarding micromotion, we consider that abutment placement and the choice of tissue-level implants can reduce the micromotion (vertically and horizontally) caused by micro-gaps and the associated marginal bone loss.

Micromotion also creates a risk of torque loss, in that loading significantly reduces the removal torque and affects the loss of the preload [20]. In our experiment, the removal torque after loading was significantly lower than that before loading, indicating a reduction in the removal torque due to loading. Furthermore, the above-mentioned experiment required a smaller torque value for tightening and a larger load. This suggests that a reduction in torque occurs even with a small load. Several studies have been conducted with loads, assuming force from posterior molars or maximum occlusal value; however, torque reduction occurs irrespective of load size. It is important to check the tightening torque regularly during maintenance, irrespective of the implantation site [7,11,14].

Care should be taken to avoid overtightening the torque during clinical maintenance. Calcaterra et al. found that re-tightening more than once may create a permanent gap between the implant abutments [21]. These findings may be explained by the mechanical wear or deformation of the component, even when tightened at the specified torque value. To prevent a decrease in pressure, in the present study, load testing was performed after the second tightening at 35 N cm. However, from the perspective of microleakage, it may have a disadvantageous effect, and there is room for debate regarding the tightening torque during maintenance.

Previous studies and the current study results indicate that the abutment sinks into the implant when the screw is tightened for implants with tapered connections, with sinkage varying by several tens of micrometers, depending on the tightening torque. Discrepancies in chair-side and laboratory-side screw fastening forces pose a risk of inaccuracies in vertical and horizontal impression precision. Such inaccuracies can result in poor alignment between the implant and the superstructure, leading to excessive stress on the structure and the potential for marginal bone resorption and implant body or prosthetic device fractures. When using implants with Morse taper connections, it is crucial to be mindful of the potential for subsidence of the implant body.

## 5. Limitations and Future Directions

The study had a few limitations. Firstly, data were obtained from experiments conducted in a laboratory under specific conditions; therefore, the results may not directly translate to implants within the body. Furthermore, although we were able to obtain novel insights into micromotion, the data obtained are limited to a type of IAC specific to the implants used. Future studies should be conducted using implants with different connections and loading conditions.

## 6. Conclusions

The present experiment was conducted to observe micromotion over time as a cause of peri-implantitis. Position data from fatigue tests were used for the measurements. As a result, within a controlled experimental environment, the temporal progression of micromotion decreased with loading over time; however, it did not cease completely. Micromotion occurs between implant abutments after superstructure placement, and there is a risk of several events in the IACs, including bacterial leakage, leakage of Ti ions, and loss of torque. Therefore, the presence of micromotion should be carefully considered when placing implants, performing prosthetic operations, and designing prosthetic devices, with the presumption it will always be present.

## Data Availability

The datasets used and analyzed during the current study are available from the corresponding author upon reasonable request.
